# Do We Produce Enough Fruits and Vegetables to Meet Global Health Need?

**DOI:** 10.1371/journal.pone.0104059

**Published:** 2014-08-06

**Authors:** Karen R. Siegel, Mohammed K. Ali, Adithi Srinivasiah, Rachel A. Nugent, K. M. Venkat Narayan

**Affiliations:** 1 Nutrition and Health Sciences, Laney Graduate School, Emory University, Atlanta, Georgia, United States of America; 2 Hubert Department of Global Health, Emory University, Atlanta, Georgia, United States of America; 3 Emory College, Emory University, Atlanta, Georgia, United States of America; 4 Department of Global Health, University of Washington, Seattle, Washington, United States of America; Universidade Federal de Vicosa, Brazil

## Abstract

**Background:**

Low fruit and vegetable (FV) intake is a leading risk factor for chronic disease globally, but much of the world’s population does not consume the recommended servings of FV daily. It remains unknown whether global supply of FV is sufficient to meet current and growing population needs. We sought to determine whether supply of FV is sufficient to meet current and growing population needs, globally and in individual countries.

**Methods and Findings:**

We used global data on agricultural production and population size to compare supply of FV in 2009 with population need, globally and in individual countries. We found that the global supply of FV falls, on average, 22% short of population need according to nutrition recommendations (supply:need ratio: 0.78 [Range: 0.05–2.01]). This ratio varies widely by country income level, with a median supply:need ratio of 0.42 and 1.02 in low-income and high-income countries, respectively. A sensitivity analysis accounting for need-side food wastage showed similar insufficiency, to a slightly greater extent (global supply:need ratio: 0.66, varying from 0.37 [low-income countries] to 0.77 [high-income countries]). Using agricultural production and population projections, we also estimated supply and need for FV for 2025 and 2050. Assuming medium fertility and projected growth in agricultural production, the global supply:need ratio for FV increases slightly to 0.81 by 2025 and to 0.88 by 2050, with similar patterns seen across country income levels. In a sensitivity analysis assuming no change from current levels of FV production, the global supply:need ratio for FV decreases to 0.66 by 2025 and to 0.57 by 2050.

**Conclusion:**

The global nutrition and agricultural communities need to find innovative ways to increase FV production and consumption to meet population health needs, particularly in low-income countries.

## Introduction

Low fruit and vegetable (FV) intake is a leading risk factor for death and disability globally, estimated to contribute to approximately 16.0 million disability-adjusted life years and 1.7 million deaths worldwide annually [1]. According to a World Health Organization report, current global dietary guidelines recommend that individuals consume at least 5 servings of FV daily [2]. Recent cross-country evidence supports this recommendation, showing a strong dose-response relationship between higher FV consumption and lower all-cause mortality [3] as well as lower risk of major chronic diseases such as cardiovascular disease, diabetes, and certain cancers, which impact every region of the world [4]–[6].

Much of the world’s population, however, does not consume the recommended five servings of FV daily. Data from 52 mainly low- and middle-income countries participating in the 2002–2003 World Health Survey reported that, overall, 77.6% of men and 78.4% of women surveyed consumed less than the recommended five daily servings of FV. The survey also showed that FV consumption patterns vary around the world, but lower-than-recommended reported consumption is common in high, middle, and low-income countries. For example, in a recent report, poor dietary habits, which includes low FV consumption, was *the* leading risk factor in the United States (U.S.), accounting for 26% of all deaths and 14% of all disability [7], and increasing individual FV consumption to up to 600 grams per day (slightly more than 5 servings per day) could reduce the total worldwide burden of disease by 1.8%, and reduce the burden of ischemic heart disease and ischemic stroke by 31% and 19% respectively [2].

Despite a wealth of research on behavioral determinants of FV, it remains unknown whether global production and supply of FV is actually sufficient to meet population needs. We used global population and agriculture databases to compare the global supply of (“supply”) with recommended dietary intake (implied “demand”, hereafter referred to as “need”) globally and in individual countries. Using agricultural production and population projections data, we also project supply and need for FV for 2025 and 2050.

## Methods

### Data Sources

We used three main data sources for our analysis: (1) Food and Agricultural Organization (FAO) 2009 Food Balance Sheets [8], (2) age-specific FV intake recommendations for individuals [2], and (3) the United Nations (UN) World Population Prospects: The 2012 Revision [9].

The FAO 2009 Food Balance Sheets (the most recent year for which these data were available) report FV (excluding wine) supply by individual country for over 175 countries. These data are calculated by taking into account production, imports and exports, and food losses (through storage, transport, and processing; feed to livestock; or use as seeds and non-dietary purposes). The data reflect “formal” food production, and do not capture FV production from subsistence farming and production, which may not enter formal economies. For the FAO Food Balance Sheets, this estimated national food supply is divided by population size estimates to derive the reported per capita supply of FV (in kg/person/year).

For FV recommendations, we used a World Health Organization (WHO) report on the quantitative comparison of different health risks worldwide [2]. The report cited previously calculated and validated estimates for the average annual weight of the 5 recommended servings of FV per day: 330 grams per day for individuals aged 0–4 years, 480 grams per day for individuals aged 5–14 years, and 600 grams per day for all individuals aged 15 years and older. We converted these data into kilograms.

The UN World Population Prospects: 2012 Revision (the most recent version) provides country-level population estimates, in terms of the total population size as well as the proportion of each country’s population by age. Calculations are done yearly using data classified by broad age groups (0–14 years, 15+ years) and for five-year periods (the latest years being 2005 and 2010) using data classified by more specific age groups, including 0–4 years, 5–14 years, and 15 years and older. To align our population estimates with age-specific FV recommendations, we used population estimates from 2010. This data source also provides population projections based on different scenarios for changing fertility levels for the period 2010–2100 for individual countries and globally.

### Data Analysis

To calculate “supply” (in kg/year), we multiplied the FAO per-capita estimates by total population estimates for each country from the UN. The equation for supply is:
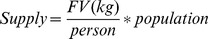



To calculate “need” (assuming all individuals are able to meet their daily recommended intake of FV – “perfect need”), we multiplied the UN’s age-specific population estimates by recommendations for FV servings per day for the same age-specific groups. Total country-specific population need (in kg/year) was then calculated by summing the recommended FV weights for all three age categories. The equation for “need” is:
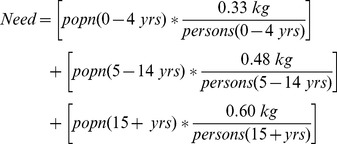



Finally, we calculated a supply:need ratio by dividing supply by need, both expressed in kg/year, where a value greater than 1.0 signifies surplus, a value of 1.0 implies balance, and less than 1.0 signifies deficit. Supply, need, and supply:need ratios were calculated for each individual country and globally. We also calculated averages of these supply, need, and supply:need ratio indicators across varying country income levels, defined according to World Bank categories: low-income economies (per capita Gross Domestic Product [GDP] of $1,025 or less), lower-middle-income economies (per capita GDP of $1,026 to $4,035), upper-middle-income economies (per capita GDP of $4,036 to $12,475), and high-income economies (per capita GDP of $12,476 or more).

For the projections for 2025 and 2050, we calculated changes in production (“supply”) using agricultural production growth rates to 2030 (1.6% for developing and 0.7% for developed countries) and 2050 (0.9% for developing and 0.3% for developed countries) as estimated by the FAO [10]. Similar to our calculations for current need, we calculated projected need by multiplying age-specific population projections for 2025 and 2050 by recommendations for FV servings per day for the same age-specific groups and summing across all three groups. For this projections analysis, we assumed a medium variant fertility scenario (2–3 children per woman).

All calculations were performed in Excel and data analysis was performed using Statistical Analysis Software (SAS) version 9.3. We used ArcMAP to illustrate the data geographically.

### Sensitivity Analyses

To account for need-side food wastage at the household/individual level, we performed a sensitivity analysis to adjust these estimates to account for wastage of 33% in high-income regions/countries and 15% in low- to middle-income regions/countries [11]. For the projections, we also performed a sensitivity analysis in order to account for “best-case” (low fertility, or <2.1 children per woman) and “worst-case” (high fertility, or >5 children per woman) scenarios [9]. In addition to the main projections analysis, we also performed a sensitivity analysis assuming current levels of agricultural production.

## Results

Table 1 shows descriptive statistics, overall and by country income level, for all countries for which all data were available (n = 170). Overall, the global supply (not including subsistence production that may not enter formal economies) of available FV falls 22% short of population’s need according to nutritional recommendations, and as much as 95% short in some countries (overall supply:need ratio: 0.78 [range: 0.05–2.01]). This ratio varies widely by country income level, with a median supply:need ratio of 0.42 in low-income countries and a median supply:need ratio of 1.02 in high-income countries (Table 1). In a sensitivity analysis in which we accounted for need-side food wastage, similarly insufficient FV supplies were noted, to a slightly greater extent. The global supply:need ratio was 0.66 when need-side wastage was accounted for, and this varied from 0.37 (low-income countries) to 0.77 (high-income countries) (see Table S1 for results by country and Table S2 for results across country income level).

**Table 1:** Descriptive Statistics of Fruit and Vegetable Supply, Need, and Supply: Need Ratio, Overall and by Country Income Level.

	*n*	Supply	Need	Supply:Need Ratio
Full Sample, all countries	170	1.15 (0.01–524.25)	1.90 (0.02–282.50)	0.78 (0.05–2.01)
Low Income	34	0.97 (0.05–7.50)	2.36 (0.13–30.18)	0.42 (0.05–0.99)
Lower-middle Income	43	1.01 (0.01–142.51)	1.49 (0.02–241.62)	0.63 (0.19–1.72)
Upper-middle Income	50	1.52 (0.01–524.25)	1.71 (0.02–282.50)	0.87 (0.24–2.01)
High Income	43	1.60 (0.04–71.63)	1.64 (0.05–64.59)	1.02 (0.55–1.86)

Notes: All numbers provided as median (range). Supply and Need are reported in billions of kilograms of fruits and vegetables. Country Income Level defined according to World Bank categories: Low-income economies ($1,025 or less), Lower-middle-income economies ($1,026 to $4,035), Upper-middle-income economies ($4,036 to $12,475), High-income economies ($12,476 or more).

The supply:need ratio also varies widely by geographical region. The highest ratios of greater than 1.0 (indicating more than sufficient supply to meet the population’s needs) are seen in the Mediterranean/North African countries of Montenegro (supply:need ratio 2.01), Greece (1.86), Turkey (1.78), Egypt (1.72), Libya (1.67), Tunisia (1.52), Italy (1.50), and Portugal (1.48); Middle Eastern countries of Iran (1.78), Israel (1.56), and Lebanon (1.46); Caribbean countries of Bahamas (1.61) and Belize (1.50); Albania (1.59); and China (1.86). The countries with the greatest shortage, where need is far greater than supply, are primarily African countries such as Eritrea (0.05), Chad (0.09), Burkina Faso (0.10), Mozambique (0.12), Ethiopia (0.12).

Table 2 shows projected supply, need and supply:need ratios overall and by country income level, for all countries for which all data was available (n = 169). Assuming medium fertility (2–3 children per woman) and projected agricultural production growth rates, the global supply:need ratio for FV increases slightly to 0.81 by 2025 and to 0.88 by 2050. As with current, the projected supply:need ratio in 2025 and 2050 varies by country income level. The lowest ratio is seen in low-income countries, where it dips to 0.30 in 2050, assuming medium fertility. The projected supply:need ratio is higher in high income countries, where it ranges from an estimated 0.98 to 1.21. In a sensitivity analysis using current levels of FV production (ie, assuming no increase in production), the global supply:need ratio for FV decreases to 0.66 by 2025 and to 0.57 by 2050 assuming medium fertility (2–3 children per woman). As with current, the projected supply:need ratio in 2025 and 2050 varies by country income level, with the lowest ratio of 0.18 in low-income countries by 2050 and the highest ratio of 0.99 in high income countries (see Table S3).

**Table 2:** Projected Need and Supply:Need Ratios, Overall and by Country Income Level.

		2025			2050		
	*n*	Supply	Need	Supply:Need Ratio	Supply	Need	Supply:Need Ratio
Full Sample, all countries	169	1.45 (0.02–675.83)			1.79 (0.02–875.25)		
High fertility			2.21 (0.02–310.96)	0.79 (0.04–2.52)		2.74 (0.02–380.34)	0.78 (0.03–3.25)
Medium fertility			2.16 (0.02–302.40)	0.81 (0.04–2.59)		2.48 (0.02–335.52)	0.88 (0.03–3.69)
Low fertility			2.10 (0.02–293.83)	0.84 (0.04–2.67)		2.23 (0.02–293.93)	1.00 (0.03–4.21)
Low Income	34	1.20 (0.07–9.67)			1.55 (0.09–12.52)		
High fertility			3.65 (0.19–37.53)	0.34 (0.04–1.15)		5.89 (0.33–48.38)	0.26 (0.03–1.30)
Medium fertility			3.55 (0.19–36.28)	0.35 (0.04–1.18)		5.28 (0.30–42.11)	0.30 (0.03–1.47)
Low fertility			3.45 (0.18–35.03)	0.36 (0.04–1.22)		4.70 (0.27–36.43)	0.33 (0.03–1.68)
Lower-middle Income	42	1.32 (0.04–183.72)			1.71 (0.06–237.93)		
High fertility			2.35 (0.04–297.58)	0.62 (0.16–1.90)		3.49 (0.05–380.34)	0.58 (0.09–2.34)
Medium fertility			2.28 (0.04–288.77)	0.64 (0.16–1.95)		3.08 (0.05–335.52)	0.66 (0.10–2.65)
Low fertility			2.21 (0.04–279.95)	0.66 (0.17–2.02)		2.70 (0.04–293.93)	0.75 (0.11–3.01)
Upper-middle Income	50	1.96 (0.02–675.83)			2.54 (0.02–875.25)		
High fertility			1.85 (0.02–310.96)	0.94 (0.19–2.52)		1.86 (0.02–327.36)	1.03 (0.12–3.25)
Medium fertility			1.79 (0.02–302.40)	0.97 (0.19–2.59)		1.64 (0.02–290.93)	1.16 (0.14–3.69)
Low fertility			1.74 (0.02–293.83)	1.00 (0.20–2.67)		1.44 (0.02–257.35)	1.33 (0.15–4.21)
High Income	43	1.79 (0.04–80.09)			1.97 (0.04–88.05)		
High fertility			1.91 (0.06–74.60)	1.04 (0.59–2.04)		2.17 (0.07–92.40)	0.98 (0.52–2.15)
Medium fertility			1.86 (0.06–72.69)	1.06 (0.61–2.09)		1.96 (0.07–83.32)	1.08 (0.58–2.38)
Low fertility			1.81 (0.06–70.79)	1.09 (0.63–2.14)		1.76 (0.06–74.67)	1.21 (0.65–2.65)

Notes: All numbers provided as median (range). Need is reported in billions of kilograms of fruits and vegetables. Country Income Level defined according to World Bank categories: Low-income economies ($1,025 or less), Lower-middle-income economies ($1,026 to $4,035), Upper-middle-income economies ($4,036 to $12,475), High-income economies ($12,476 or more). Fertility is defined according to the United Nations World Population Prospects, 2012 Revision: high fertility (more than 5 children per woman), medium fertility (2–3 children per woman), and low fertility (less than 2.1 children per woman).

**Figure 1:**
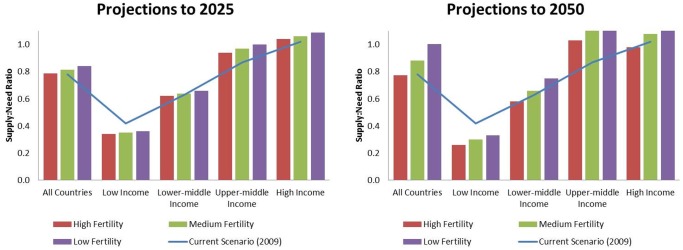
Projected Supply: Need Ratio, 2025 and 2050. Notes: Country Income Level defined according to World Bank categories: Low-income economies ($1,025 or less), Lower-middle-income economies ($1,026 to $4,035), Upper-middle-income economies ($4,036 to $12,475), High-income economies ($12,476 or more). Fertility is defined according to the United Nations World Population Prospects, 2012 Revision: high fertility (5 or more children per woman), medium fertility (2–3 children per woman), and low fertility (less than 2.1 children per woman).

Figure 1 illustrates current and projected supply:need ratios, highlighting the growing gap between supply and need in low-income countries over time.

## Discussion

Within the formal agricultural sector, there is an estimated 22% supply gap in meeting current need for FV (34% when considering food wastage at the household/individual level), and this varies from 58% to 13% across low- and upper-middle income countries. High income countries appear to have sufficient supply (supply:need ratio is 1.02). Furthermore, these gaps between high/middle-income and low-income countries will worsen with time. Assuming medium fertility and projected increases in production of FV, the global supply:need ratio for FV increases slightly to 0.81 by 2025 and to 0.88 by 2050, but divergence occurs whereby we estimated a supply gap of 70% and 65% in low-income countries by 2025 and 2050, respectively, while middle- and high-income countries approach a supply:need of 1.0, implying balance of supply and need. Without the projected increase in FV production, however, the global supply:need ratio could decrease to 0.66 by 2025 and to 0.57 by 2050, dipping as low as 0.18 in low-income countries.

There may be several reasons for these findings. Supply-side factors include subsidies and distribution systems for supply, and international trade for addressing imbalances in supply:need ratios across countries and country-income levels [12]. Many countries provide producer-end subsidies for grain crops and meat/dairy, incentivizing farmers to grow these items while dis-incentivizing FV production. In the U.S., the commodity crops receiving the largest amount of agricultural subsidies are grains, livestock, and dairy and under current agricultural policy, farmers are penalized for growing “specialty crops” (FV) if they have received federal farm payments to grow other crops [13], [14]. As a result, grains, meat, and dairy are abundant [15], the supply of FV, at least in the US, is insufficient to meet population needs [16]. In low-income countries, where we found FV need to be greatest, the lack of adequate distribution systems may lead to supply-side wastage and disincentives for their production. This is an issue particularly in warm climates like India and Africa, where FV are prone to spoiling before reaching their market destinations [17].

In particular, international trade (and climates ideal for growing FV) could help explain the differences in findings across country-income groups and geographical regions. International trade in FV, which since the 1980s has expanded more rapidly than other agricultural commodities and was 17% of total agricultural trade in 2001, is also an important consideration for increasing supply of FV, particularly in countries where production may be high but supply low due to exports [18]. Climates ideal for growing FV is also a very important supply-side factor when considering FV production. As noted in the results section, there appear to be varying levels of agronomical potentials of countries located in different geographical regions, as highlighted by the large geographical variations in the supply:need ratio, with high ratios seen in many Mediterranean countries. For example, it is known that Mediterranean countries are great producers of fruits for the fresh market due to climatic conditions – drip irrigation combined with dry summers is a perfect scenario for producing high quality crops (although a substantial proportion of this production is exported to other countries).

On the need side of the equation, population size – and relatively large projected increases, particularly in certain low-income countries – helps to explain the large and growing gaps between supply and need in these countries. The projections data show that, assuming an estimated increase in FV production, the supply:need ratio narrows on a global scale, but that it widens to a considerable extent in low-income countries, primarily as a reflection of higher fertility in these countries and agricultural production growth rates that cannot keep up with population growth. The ability to produce enough FV to meet the needs of large and growing populations, coupled with the supply-side limitations mentioned above, are of particular concern for these countries. In the 18^th^ century, Malthus projected that human population growth would outpace expansion in food production. Since then, with the help of technological advances spurred by the Green Revolution, production and subsequent supply of carbohydrates and grains has increased to meet global population needs. Our projections analysis suggests that high-income countries may be making strides towards increasing production and subsequent supply of FV to meet their population’s needs, but that the same cannot be said for the low-income countries, at least within the formal agricultural economy, where the gap in supply not taking subsistence farming into account could widen to 65% by 2050 if not addressed. Of greater concern, if projected increases in agricultural production of FV do not manifest, by 2025 and 2050 high- and low-income countries alike may not able to meet their population’s needs for FV.

While ecological data suggests that food availability can influence food consumption patterns and in turn, cardiometabolic health outcomes like diabetes [19], [20], to date there has been a relatively limited focus on production and supply of FV. Researchers at America’s Farmland Trust investigated supply of FV in the United States (U.S.) alone; they concluded that an estimated 13 million more acres of farmland would be needed to produce a sufficient supply for the U.S. population [21]. Our analysis builds upon these results. The first study to incorporate empirical country-level data and age-specific recommendations for FV consumption to examine global and country-specific FV supply (in the formal sector) as it compares to need, our study highlights inadequate supply of FV as it compares to the population’s nutritional needs, from the perspective of preventing chronic diseases, which currently place enormous burdens on countries around the world and are largely preventable through healthy diet and higher FV consumption [2], [7].

These findings must be contextualized by limitations to our analysis. First, the data used were macro-level indicators collected at the country-level and may be prone to either over- or under-estimation. The data do not account for how much people actually access FV in various countries nor the quality and diversity of FV consumption, including how these FV are consumed (raw, cooked, or processed FV have different nutrient bioavailability), nor how much *individuals* actually consume. For example, many Mediterranean and Caribbean countries, which were found to have high supply:need ratios, are great citrus producers, but in the latter fruits are processed (for juice) and not sold on the fresh market. Additionally, every fruit and vegetable does not have the same macro- and micro-nutrient content, and even the same fruit or vegetable grown in a different climate or soil may have differing amounts of macro- and micro-nutrients. Additionally, there may also be differences in the quality and validity of the data in high- versus low-income countries. However, the FAO Food Balance Sheets are the most commonly used source of food availability information at the national level, providing standardized estimates of the average amount of food available per person on a daily basis and a useful tool for international comparisons [22]. Second, our analysis is at the country level, and therefore does not take into account urban/rural differences in supply that may result from challenges in distribution (for example, transporting FV from the farm to urban areas. This may be a particular issue in resource-poor settings, where distributional infrastructure may be lacking. Further analyses could investigate these issues, analyzing potential heterogeneity of supply and need within countries and in urban versus rural settings.

A third limitation is that our analysis does not capture local food economies (ie, subsistence farming and food production) in individual countries. That is, it does not take into account the production of FV that may exist outside of the formal agricultural sector (i.e., home gardens), which may vary widely across countries. This may be an additional area of future research. For example, researchers could utilize the powerful technologies of Google Earth to look within countries, at the regional, city, district, or even household level, at the presence or absence of informal community or household gardens. Lastly, our analysis does not incorporate additional economic indicators such as the costs of production or the resulting prices of FV. Our results suggest that insufficient supply exists relative to population needs under current production conditions. We have not taken into account the potential for supply to increase due to technological improvements and supportive government policies. Both those factors could lower FV prices and increase consumption.

Our study adds unique value by underlining the importance of increasing supply of FV and sets the stage for further analyses to delve further into the policy levers for increasing production and supply. In particular, investigating the supply of FV resulting from subsistence farming could augment our analysis. At the same time, continuing efforts to improve demand for FV – for example, through public health education and health promotion programs, proposing taxes on foods of low nutritional value (e.g., soda, high-fat foods) or subsidies on foods of high nutrition value (e.g., FV), improved food labeling, and stricter controls on the marketing of foods [23]–[27] – is equally important. Without an accompanying increase in supply, however, these efforts may have limited reach. It is hoped that our straightforward analysis, highlighting inadequate formal supply of FV in the context of perfect need (assuming all individuals are able to meet their daily recommended intake of FV), may provide value by offering an understanding of the current and future global disconnect between nutritional recommendations and supply of FV, and guide conversations and future investigations to consider appropriate policy responses. The triumph of grains production over the doom and gloom forecast of Malthus is a major testament to the technological and organizational success of food production and distribution worldwide that has accompanied industrialization and modern development. The current state of affairs presents a challenge to the global nutrition and agricultural communities to increase FV production in the same way, especially in low-income countries. Change is possible.

## Supporting Information

Table S1 **List of Countries and Their Respective Supply, Need, and Supply:Need Ratios.** Notes: All numbers provided as median (range). Need is reported in billions of kilograms of fruits and vegetables. Country Income Level defined according to World Bank categories: Low-income economies ($1,025 or less), Lower-middle-income economies ($1,026 to $4,035), Upper-middle-income economies ($4,036 to $12,475), High-income economies ($12,476 or more). Fertility is defined according to the United Nations World Population Prospects, 2012 Revision: high fertility (more than 5 children per woman), medium fertility (2–3 children per woman), and low fertility (less than 2.1 children per woman).
(DOCX)

Table S2 **Sensitivity Analysis of Fruit and Vegetable Supply, Need, and Supply:Need Ratio, Overall and by Country Income Level.** Notes: All numbers provided as median (range). Supply and Need are reported in billions of kilograms of fruits and vegetables. Country Income Level defined according to World Bank categories: Low-income economies ($1,025 or less), Lower-middle-income economies ($1,026 to $4,035), Upper-middle-income economies ($4,036 to $12,475), High-income economies ($12,476 or more).
(DOCX)

Table S3 **Sensitivity Analysis of Projected Need and Supply:Need Ratios (Assuming Current Levels of Agricultural Production), Overall and by Country Income Level.** Notes: All numbers provided as median (range). Need is reported in billions of kilograms of fruits and vegetables. Country Income Level defined according to World Bank categories: Low-income economies ($1,025 or less), Lower-middle-income economies ($1,026 to $4,035), Upper-middle-income economies ($4,036 to $12,475), High-income economies ($12,476 or more). Fertility is defined according to the United Nations World Population Prospects, 2012 Revision: high fertility (more than 5 children per woman), medium fertility (2–3 children per woman), and low fertility (less than 2.1 children per woman).
(DOCX)
