# Supplementary material for: Do We Produce Enough Fruits and Vegetables to Meet Global Health Need?
Source: PLoS One. 2014 Aug 6;9(8):e104059. doi: 10.1371/journal.pone.0104059 (PMC4123909; doi:10.1371/journal.pone.0104059)
Supplement: Table S1 — List of Countries and Their Respective Supply, Need, and Supply:Need Ratios. Notes: All numbers provided as median (range). Need is reported in billions of kilograms of fruits and vegetables. Country Income Level defined according to World Bank categories: Low-income economies ($1,025 or less), Lower-middle-income economies ($1,026 to $4,035), Upper-middle-income economies ($4,036 to $12,475), High-income economies ($12,476 or more). Fertility is defined according to the United Nations World Population Prospects, 2012 Revision: high fertility (more than 5 children per woman), medium fertility (2–3 children per woman), and low fertility (less than 2.1 children per woman). (DOCX) [file pone.0104059.s001.docx]

**Table S1: List of Countries and Their Respective Supply, Need, and Supply:Need Ratios**

| **Country** | **Supply** | **Not Accounting for Need-side Food Wastage** | | **Accounting for Need-side**  **Food Wastage** | |
| --- | --- | --- | --- | --- | --- |
|  |  | **Need** | **Supply:Need Ratio** | **Need** | **Supply:Need Ratio** |
| Albania | 1.03 | 0.65 | 1.59 | 0.75 | 1.39 |
| Algeria | 7.59 | 7.46 | 1.02 | 8.58 | 0.88 |
| Angola | 0.87 | 3.67 | 0.24 | 4.22 | 0.21 |
| Antigua and Barbuda | 0.02 | 0.02 | 1.31 | 0.02 | 1.14 |
| Argentina | 5.66 | 8.22 | 0.69 | 9.46 | 0.60 |
| Australia | 4.39 | 4.64 | 0.95 | 6.18 | 0.71 |
| Austria | 1.91 | 1.77 | 1.08 | 2.35 | 0.81 |
| Azerbaijan | 2.09 | 1.86 | 1.12 | 2.14 | 0.98 |
| Bahamas | 0.12 | 0.07 | 1.61 | 0.10 | 1.21 |
| Bangladesh | 7.50 | 30.18 | 0.25 | 34.71 | 0.22 |
| Barbados | 0.06 | 0.06 | 0.99 | 0.08 | 0.74 |
| Belarus | 2.04 | 1.99 | 1.02 | 2.29 | 0.89 |
| Belgium | 2.36 | 2.28 | 1.03 | 3.04 | 0.78 |
| Belize | 0.09 | 0.06 | 1.50 | 0.07 | 1.30 |
| Benin | 0.78 | 1.82 | 0.43 | 2.09 | 0.38 |
| Bolivia | 1.01 | 2.00 | 0.51 | 2.30 | 0.44 |
| Bosnia and Herzegovina | 1.13 | 0.80 | 1.41 | 0.93 | 1.22 |
| Botswana | 0.15 | 0.39 | 0.39 | 0.45 | 0.34 |
| Brazil | 32.50 | 39.77 | 0.82 | 45.73 | 0.71 |
| Brunei Darussalam | 0.06 | 0.08 | 0.76 | 0.11 | 0.57 |
| Bulgaria | 0.80 | 1.56 | 0.51 | 1.79 | 0.44 |
| Burkina Faso | 0.31 | 2.94 | 0.10 | 3.38 | 0.09 |
| Burundi | 1.53 | 1.75 | 0.87 | 2.02 | 0.76 |
| Cambodia | 0.82 | 2.86 | 0.29 | 3.29 | 0.25 |
| Cameroon | 3.98 | 3.94 | 1.01 | 4.53 | 0.88 |
| Canada | 8.31 | 7.13 | 1.17 | 9.48 | 0.88 |
| Cape Verde | 0.07 | 0.10 | 0.72 | 0.11 | 0.63 |
| Central African Republic | 0.29 | 0.84 | 0.34 | 0.97 | 0.30 |
| Chad | 0.20 | 2.19 | 0.09 | 2.52 | 0.08 |
| Chile | 2.66 | 3.53 | 0.76 | 4.05 | 0.66 |
| China | 524.25 | 282.50 | 1.86 | 324.88 | 1.61 |
| Colombia | 6.82 | 9.35 | 0.73 | 10.75 | 0.63 |
| Comoros | 0.08 | 0.13 | 0.61 | 0.15 | 0.53 |
| Congo, Dem. Rep. | 5.51 | 11.78 | 0.47 | 13.54 | 0.41 |
| Costa Rica | 0.61 | 0.95 | 0.64 | 1.10 | 0.56 |
| Cote d'Ivoire | 2.08 | 3.65 | 0.57 | 4.20 | 0.50 |
| Croatia | 0.90 | 0.91 | 0.99 | 1.21 | 0.75 |
| Cuba | 3.04 | 2.36 | 1.29 | 2.71 | 1.12 |
| Cyprus | 0.25 | 0.23 | 1.07 | 0.31 | 0.80 |
| Czech Republic | 1.60 | 2.22 | 0.72 | 2.95 | 0.54 |
| Denmark | 1.30 | 1.15 | 1.13 | 1.54 | 0.85 |
| Djibouti | 0.07 | 0.16 | 0.40 | 0.19 | 0.34 |
| Dominican Republic | 2.42 | 2.00 | 1.21 | 2.30 | 1.05 |
| Ecuador | 2.49 | 3.00 | 0.83 | 3.45 | 0.72 |
| Egypt | 26.67 | 15.54 | 1.72 | 17.87 | 1.49 |
| El Salvador | 0.88 | 1.24 | 0.71 | 1.43 | 0.61 |
| Eritrea | 0.05 | 1.10 | 0.05 | 1.26 | 0.04 |
| Estonia | 0.25 | 0.27 | 0.91 | 0.36 | 0.68 |
| Ethiopia | 2.03 | 16.63 | 0.12 | 19.13 | 0.11 |
| Fiji | 0.07 | 0.17 | 0.41 | 0.20 | 0.36 |
| Finland | 0.92 | 1.12 | 0.82 | 1.49 | 0.62 |
| France | 13.46 | 13.13 | 1.02 | 17.47 | 0.77 |
| French Polynesia | 0.04 | 0.06 | 0.72 | 0.07 | 0.54 |
| Gabon | 0.28 | 0.30 | 0.92 | 0.35 | 0.80 |
| Gambia | 0.06 | 0.32 | 0.20 | 0.37 | 0.18 |
| Georgia | 0.45 | 0.91 | 0.49 | 1.05 | 0.43 |
| Germany | 14.43 | 17.52 | 0.82 | 23.30 | 0.62 |
| Ghana | 4.74 | 4.71 | 1.01 | 5.42 | 0.88 |
| Greece | 4.35 | 2.33 | 1.86 | 3.10 | 1.40 |
| Grenada | 0.02 | 0.02 | 0.91 | 0.02 | 0.79 |
| Guatemala | 1.75 | 2.76 | 0.63 | 3.18 | 0.55 |
| Guinea | 1.48 | 2.08 | 0.71 | 2.39 | 0.62 |
| Guinea-Bissau | 0.10 | 0.30 | 0.34 | 0.35 | 0.30 |
| Guyana | 0.08 | 0.15 | 0.51 | 0.18 | 0.44 |
| Haiti | 0.91 | 1.94 | 0.47 | 2.24 | 0.41 |
| Honduras | 1.12 | 1.49 | 0.75 | 1.72 | 0.65 |
| Hungary | 2.19 | 2.10 | 1.04 | 2.80 | 0.78 |
| Iceland | 0.06 | 0.07 | 0.95 | 0.09 | 0.72 |
| India | 142.51 | 241.62 | 0.59 | 277.87 | 0.51 |
| Indonesia | 25.55 | 48.22 | 0.53 | 55.46 | 0.46 |
| Iran, Islamic Rep. | 27.07 | 15.19 | 1.78 | 17.47 | 1.55 |
| Ireland | 1.09 | 0.92 | 1.19 | 1.22 | 0.89 |
| Israel | 2.34 | 1.50 | 1.56 | 1.99 | 1.17 |
| Italy | 18.80 | 12.73 | 1.48 | 16.93 | 1.11 |
| Jamaica | 0.52 | 0.55 | 0.93 | 0.64 | 0.81 |
| Japan | 19.68 | 26.87 | 0.73 | 35.74 | 0.55 |
| Jordan | 0.90 | 1.27 | 0.71 | 1.46 | 0.62 |
| Kazakhstan | 3.45 | 3.23 | 1.07 | 3.71 | 0.93 |
| Kenya | 4.38 | 7.83 | 0.56 | 9.01 | 0.49 |
| Kiribati | 0.01 | 0.02 | 0.65 | 0.02 | 0.57 |
| Korea, Dem. Rep. | 4.98 | 5.03 | 0.99 | 5.79 | 0.86 |
| Kuwait | 0.63 | 0.61 | 1.04 | 0.81 | 0.78 |
| Kyrgyz Republic | 1.00 | 1.07 | 0.94 | 1.23 | 0.82 |
| Lao PDR | 1.17 | 1.25 | 0.94 | 1.44 | 0.81 |
| Latvia | 0.34 | 0.44 | 0.78 | 0.51 | 0.68 |
| Lebanon | 1.30 | 0.89 | 1.46 | 1.02 | 1.27 |
| Lesotho | 0.08 | 0.39 | 0.19 | 0.45 | 0.17 |
| Liberia | 0.26 | 0.76 | 0.34 | 0.87 | 0.30 |
| Libya | 2.02 | 1.21 | 1.67 | 1.39 | 1.45 |
| Lithuania | 0.57 | 0.64 | 0.89 | 0.74 | 0.78 |
| Luxembourg | 0.14 | 0.11 | 1.30 | 0.14 | 0.98 |
| Macedonia, FYR | 0.61 | 0.44 | 1.39 | 0.50 | 1.21 |
| Madagascar | 1.17 | 4.03 | 0.29 | 4.64 | 0.25 |
| Malawi | 1.18 | 2.84 | 0.41 | 3.27 | 0.36 |
| Malaysia | 2.60 | 5.72 | 0.45 | 6.58 | 0.40 |
| Maldives | 0.09 | 0.07 | 1.31 | 0.08 | 1.14 |
| Mali | 1.34 | 2.63 | 0.51 | 3.03 | 0.44 |
| Malta | 0.13 | 0.09 | 1.42 | 0.12 | 1.07 |
| Mauritania | 0.15 | 0.70 | 0.22 | 0.80 | 0.19 |
| Mauritius | 0.16 | 0.25 | 0.64 | 0.29 | 0.55 |
| Mexico | 18.63 | 23.65 | 0.79 | 27.20 | 0.69 |
| Moldova | 0.56 | 0.75 | 0.75 | 0.86 | 0.66 |
| Mongolia | 0.17 | 0.55 | 0.32 | 0.63 | 0.28 |
| Montenegro | 0.26 | 0.13 | 2.01 | 0.15 | 1.75 |
| Morocco | 6.92 | 6.38 | 1.09 | 7.34 | 0.94 |
| Mozambique | 0.54 | 4.54 | 0.12 | 5.23 | 0.10 |
| Myanmar | 6.25 | 10.55 | 0.59 | 12.13 | 0.52 |
| Namibia | 0.10 | 0.43 | 0.24 | 0.49 | 0.21 |
| Nepal | 3.78 | 5.27 | 0.72 | 6.06 | 0.62 |
| Netherlands | 3.52 | 3.46 | 1.02 | 4.61 | 0.76 |
| New Caledonia | 0.04 | 0.05 | 0.71 | 0.07 | 0.53 |
| New Zealand | 1.09 | 0.90 | 1.21 | 1.20 | 0.91 |
| Nicaragua | 0.33 | 1.15 | 0.28 | 1.32 | 0.25 |
| Niger | 0.93 | 2.96 | 0.31 | 3.40 | 0.27 |
| Nigeria | 17.41 | 30.38 | 0.57 | 34.94 | 0.50 |
| Norway | 1.00 | 1.01 | 0.99 | 1.35 | 0.74 |
| Pakistan | 11.34 | 34.09 | 0.33 | 39.20 | 0.29 |
| Panama | 0.42 | 0.74 | 0.57 | 0.85 | 0.49 |
| Paraguay | 0.82 | 1.28 | 0.64 | 1.47 | 0.56 |
| Peru | 4.28 | 5.87 | 0.73 | 6.75 | 0.63 |
| Philippines | 16.87 | 18.42 | 0.92 | 21.18 | 0.80 |
| Poland | 6.96 | 8.01 | 0.87 | 10.66 | 0.65 |
| Portugal | 3.33 | 2.22 | 1.50 | 2.96 | 1.13 |
| Republic of Korea | 14.39 | 10.15 | 1.42 | 13.50 | 1.07 |
| Romania | 4.87 | 4.59 | 1.06 | 5.27 | 0.92 |
| Russian Federation | 26.49 | 30.10 | 0.88 | 34.61 | 0.77 |
| Rwanda | 2.02 | 2.06 | 0.98 | 2.37 | 0.85 |
| Samoa | 0.04 | 0.04 | 1.08 | 0.04 | 0.94 |
| Sao Tome and Principe | 0.04 | 0.03 | 1.23 | 0.04 | 1.07 |
| Saudi Arabia | 4.46 | 5.44 | 0.82 | 7.24 | 0.62 |
| Senegal | 1.01 | 2.47 | 0.41 | 2.84 | 0.35 |
| Serbia | 1.75 | 2.02 | 0.87 | 2.32 | 0.75 |
| Seychelles | 0.01 | 0.02 | 0.67 | 0.02 | 0.58 |
| Sierra Leone | 0.48 | 1.10 | 0.44 | 1.27 | 0.38 |
| Slovak Republic | 0.92 | 1.14 | 0.80 | 1.52 | 0.60 |
| Slovenia | 0.46 | 0.43 | 1.06 | 0.57 | 0.80 |
| Solomon Islands | 0.04 | 0.10 | 0.39 | 0.12 | 0.34 |
| South Africa | 3.83 | 10.30 | 0.37 | 11.85 | 0.32 |
| Spain | 10.64 | 9.68 | 1.10 | 12.88 | 0.83 |
| Sri Lanka | 1.44 | 4.22 | 0.34 | 4.85 | 0.30 |
| St. Lucia | 0.02 | 0.04 | 0.69 | 0.04 | 0.60 |
| St. Vincent and the Grenadines | 0.02 | 0.02 | 1.04 | 0.03 | 0.91 |
| Sudan | 3.88 | 6.85 | 0.57 | 7.88 | 0.49 |
| Suriname | 0.07 | 0.11 | 0.65 | 0.12 | 0.56 |
| Swaziland | 0.09 | 0.23 | 0.40 | 0.27 | 0.35 |
| Sweden | 1.93 | 1.96 | 0.99 | 2.60 | 0.74 |
| Switzerland | 1.51 | 1.64 | 0.92 | 2.19 | 0.69 |
| Syrian Arab Republic | 4.88 | 4.24 | 1.15 | 4.87 | 1.00 |
| Tajikistan | 1.18 | 1.50 | 0.79 | 1.72 | 0.69 |
| Tanzania | 4.85 | 8.53 | 0.57 | 9.81 | 0.49 |
| Thailand | 10.58 | 13.77 | 0.77 | 15.84 | 0.67 |
| Timor-Leste | 0.04 | 0.20 | 0.19 | 0.23 | 0.16 |
| Togo | 0.19 | 1.21 | 0.16 | 1.39 | 0.13 |
| Trinidad and Tobago | 0.15 | 0.27 | 0.55 | 0.36 | 0.41 |
| Tunisia | 3.29 | 2.17 | 1.52 | 2.50 | 1.32 |
| Turkey | 25.95 | 14.62 | 1.78 | 16.81 | 1.54 |
| Turkmenistan | 0.93 | 1.01 | 0.92 | 1.16 | 0.80 |
| Uganda | 5.95 | 6.36 | 0.94 | 7.31 | 0.81 |
| Ukraine | 9.41 | 9.68 | 0.97 | 11.13 | 0.85 |
| United Arab Emirates | 1.69 | 1.77 | 0.95 | 2.35 | 0.72 |
| United Kingdom | 13.26 | 12.91 | 1.03 | 17.17 | 0.77 |
| United States | 71.63 | 64.59 | 1.11 | 85.91 | 0.83 |
| Uruguay | 0.43 | 0.69 | 0.62 | 0.80 | 0.54 |
| Uzbekistan | 7.65 | 5.57 | 1.37 | 6.40 | 1.20 |
| Vanuatu | 0.03 | 0.05 | 0.76 | 0.05 | 0.66 |
| Venezuela | 3.48 | 5.83 | 0.60 | 6.70 | 0.52 |
| Vietnam | 12.38 | 18.20 | 0.68 | 20.93 | 0.59 |
| Yemen | 1.94 | 4.39 | 0.44 | 5.05 | 0.39 |
| Zambia | 0.52 | 2.49 | 0.21 | 2.87 | 0.18 |
| Zimbabwe | 0.39 | 2.52 | 0.15 | 2.90 | 0.13 |

Note: Kiribati is not included in the projections analysis due to unavailable data. The columns accounting for need-side food wastage incorporate factors of 15% and 33% wastage in low/middle-income and high-income countries, respectively. The columns not accounting for need-side food wastage do not incorporate these wastage factors. Supply-side wastage is included in all estimates.
