# Supplementary material for: Do We Produce Enough Fruits and Vegetables to Meet Global Health Need?
Source: PLoS One. 2014 Aug 6;9(8):e104059. doi: 10.1371/journal.pone.0104059 (PMC4123909; doi:10.1371/journal.pone.0104059)
Supplement: Table S2 — Sensitivity Analysis of Fruit and Vegetable Supply, Need, and Supply:Need Ratio, Overall and by Country Income Level. Notes: All numbers provided as median (range). Supply and Need are reported in billions of kilograms of fruits and vegetables. Country Income Level defined according to World Bank categories: Low-income economies ($1,025 or less), Lower-middle-income economies ($1,026 to $4,035), Upper-middle-income economies ($4,036 to $12,475), High-income economies ($12,476 or more). (DOCX) [file pone.0104059.s002.docx]

Table S2: Sensitivity Analysis of Fruit and Vegetable Supply, Need, and Supply:Need Ratio, Overall and by Country Income Level

|  | ***n*** | **Supply** | **Need** | **Supply:Need Ratio** |
| --- | --- | --- | --- | --- |
| Full Sample, all countries | 170 | 1.15 (0.01 – 524.25) | 2.29 (0.02 – 324.88) | 0.66 (0.04 – 1.75) |
| Low Income | 34 | 0.97 (0.05 – 7.50) | 2.71 (0.15 – 34.71) | 0.37 (0.04 – 0.86) |
| Lower-middle Income | 43 | 1.01 (0.01 – 142.51) | 1.72 (0.02 – 277.87) | 0.55 (0.16 – 1.49) |
| Upper-middle Income | 50 | 1.52 (0.01 – 524.25) | 1.97 (0.02 – 324.88) | 0.76 (0.21 – 1.75) |
| High Income | 43 | 1.60 (0.04 – 71.63) | 2.19 (0.07 – 85.91) | 0.77 (0.41 – 1.40) |

Notes: All numbers provided as median (range). Supply and Need are reported in billions of kilograms of fruits and vegetables. Country Income Level defined according to World Bank categories: Low-income economies ($1,025 or less), Lower-middle-income economies ($1,026 to $4,035), Upper-middle-income economies ($4,036 to $12,475), High-income economies ($12,476 or more).
