# Supplementary material for: Do We Produce Enough Fruits and Vegetables to Meet Global Health Need?
Source: PLoS One. 2014 Aug 6;9(8):e104059. doi: 10.1371/journal.pone.0104059 (PMC4123909; doi:10.1371/journal.pone.0104059)
Supplement: Table S3 — Sensitivity Analysis of Projected Need and Supply:Need Ratios (Assuming Current Levels of Agricultural Production), Overall and by Country Income Level. Notes: All numbers provided as median (range). Need is reported in billions of kilograms of fruits and vegetables. Country Income Level defined according to World Bank categories: Low-income economies ($1,025 or less), Lower-middle-income economies ($1,026 to $4,035), Upper-middle-income economies ($4,036 to $12,475), High-income economies ($12,476 or more). Fertility is defined according to the United Nations World Population Prospects, 2012 Revision: high fertility (more than 5 children per woman), medium fertility (2–3 children per woman), and low fertility (less than 2.1 children per woman). (DOCX) [file pone.0104059.s003.docx]

Table S3: Sensitivity Analysis of Projected Need and Supply:Need Ratios (Assuming Current Levels of Agricultural Production), Overall and by Country Income Level

|  |  | **2025** | | **2050** | |
| --- | --- | --- | --- | --- | --- |
|  | ***n*** | **Need** | **Supply:Need Ratio** | **Need** | **Supply:Need Ratio** |
| Full Sample, all countries | 169 |  |  |  |  |
| High fertility |  | 2.21 (0.02 – 310.96) | 0.64 (0.03 – 1.95) | 2.74 (0.02 – 380.34) | 0.50 (0.02 – 1.95) |
| Medium fertility |  | 2.16 (0.02 – 302.40) | 0.66 (0.03 – 2.01) | 2.48 (0.02 – 335.52) | 0.57 (0.02 – 2.21) |
| Low fertility |  | 2.10 (0.02 – 293.83) | 0.68 (0.03 – 2.07) | 2.23 (0.02 – 293.93) | 0.65 (0.02 – 2.52) |
| Low Income | 34 |  |  |  |  |
| High fertility |  | 3.65 (0.19 – 37.53) | 0.27 (0.03 – 0.89) | 5.89 (0.33 – 48.38) | 0.16 (0.02 – 0.78) |
| Medium fertility |  | 3.55 (0.19 – 36.28) | 0.28 (0.03 – 0.92) | 5.28 (0.30 – 42.11) | 0.18 (0.02 – 0.88) |
| Low fertility |  | 3.45 (0.18 – 35.03) | 0.29 (0.03 – 0.94) | 4.70 (0.27 – 36.43) | 0.20 (0.02 – 1.01) |
| Lower-middle Income | 42 |  |  |  |  |
| High fertility |  | 2.35 (0.04 – 297.58) | 0.48 (0.12 – 1.47) | 3.49 (0.05 – 380.34) | 0.35 (0.05 – 1.40) |
| Medium fertility |  | 2.28 (0.04 – 288.77) | 0.50 (0.13 – 1.52) | 3.08 (0.05 – 335.52) | 0.39 (0.06 – 1.58) |
| Low fertility |  | 2.21 (0.04 – 279.95) | 0.51 (0.13 – 1.56) | 2.70 (0.04 – 293.93) | 0.45 (0.07 – 1.80) |
| Upper-middle Income | 50 |  |  |  |  |
| High fertility |  | 1.85 (0.02 – 310.96) | 0.76 (0.15 – 1.95) | 1.86 (0.02 – 327.36) | 0.69 (0.07 – 1.95) |
| Medium fertility |  | 1.79 (0.02 – 302.40) | 0.79 (0.15 – 2.01) | 1.64 (0.02 – 290.93) | 0.78 (0.08 – 2.21) |
| Low fertility |  | 1.74 (0.02 – 293.83) | 0.81 (0.15 – 2.07) | 1.44 (0.02 – 257.35) | 0.88 (0.09 – 2.52) |
| High Income | 43 |  |  |  |  |
| High fertility |  | 1.91 (0.06 – 74.60) | 0.93 (0.53 – 1.83) | 2.17 (0.07 – 92.40) | 0.79 (0.42 – 1.75) |
| Medium fertility |  | 1.86 (0.06 – 72.69) | 0.95 (0.54 – 1.87) | 1.96 (0.07 – 83.32) | 0.88 (0.47 – 1.94) |
| Low fertility |  | 1.81 (0.06 – 70.79) | 0.98 (0.56 – 1.92) | 1.76 (0.06 – 74.67) | 0.99 (0.53 – 2.16) |

Notes: All numbers provided as median (range). Need is reported in billions of kilograms of fruits and vegetables. Country Income Level defined according to World Bank categories: Low-income economies ($1,025 or less), Lower-middle-income economies ($1,026 to $4,035), Upper-middle-income economies ($4,036 to $12,475), High-income economies ($12,476 or more). Fertility is defined according to the United Nations World Population Prospects, 2012 Revision: high fertility (more than 5 children per woman), medium fertility (2-3 children per woman), and low fertility (less than 2.1 children per woman.
